# Supplementary material for: A Systematic Review of the Efficacy of Preclinical Models of Lung Cancer Drugs
Source: Front Oncol. 2020 Apr 23;10:591. doi: 10.3389/fonc.2020.00591 (PMC7190806; doi:10.3389/fonc.2020.00591)
Supplement: Supplementary file 1 [file Table_1.DOCX]

**Supplemental Table 1. Mechanism-Based Categorization of Drugs**

| **Nucleic acid damaging agents** | |
| --- | --- |
| Failed Drugs (n=32) | Approved Drugs (n=11) |
| 4-Ipomeanol | Mustargen (Mechlorethamine Hydrochloride) |
| Canfosfamide (TER 286) | Abitrexate (Methotrexate) |
| Glufosfamide | Alimta (Pemetrexed Disodium) |
| PR 104 | Gemzar (Gemcitabine Hydrochloride) |
| Temozolomide | Doxorubicin Hydrochloride |
| Diflomotecan (BN80915) | Etoposide |
| OSI 211 (liposomal lurtotecan), NX211 | Abraxane (Paclitaxel Albumin-stabilized Nanoparticle Formulation) |
| Pegamotecan (Enzon) | Navelbine (Vinorelbine Tartrate) |
| Rubitecan | Taxol (Paclitaxel) |
| Vosaroxin | Taxotere (Docetaxel) |
| DMDC | Carboplatin |
| Gemcitabine elaidate (CO-101 and CP-4126) |  |
| Tezacitabine |  |
| BMS 184476 |  |
| BMS 275183 |  |
| Fosbretabulin (CA4P) |  |
| KOS 862 (Epothilone D) |  |
| Milataxel (MAC-321) |  |
| Mivobulin (NSC 370147) |  |
| Rhizoxin |  |
| Gimeracil/oteracil/tegafur (S-1) |  |
| Imetelstat |  |
| ABT 751 |  |
| BBR 3464 (Triplatin Tetranitrate) |  |
| Chlorosulfaquinoxaline (CQS) |  |
| ILX 295501 |  |
| Lometrexol |  |
| Nolatrexed |  |
| OCX 191 (triapine) |  |
| Picoplatin |  |
| Rabusertib |  |
| SGN 15 (BR96-Doxorubicin) DNA damaging |  |
| **Tumor acting Cell signaling interrupting agents** | |
| Failed Drugs (n=67) | Approved Drugs (n=14) |
| ABT 510 | Iressa (Gefitinib) |
| BMS 181174 | Mekinist (Trametinib) and tafinlar (combination) |
| Efatutazone | Keytruda (Pembrolizumab) PD-1 inhibitor cell signaling |
| Enzastaurin | Opdivo (Nivolumab) PD-1 inhibitor cell signaling |
| Epidermal growth factor fusion toxin (DAB389EGF) | Portrazza (Necitumumab) EGFR inhibitor cell signaling |
| Etalocib (LY293111) | Tecentriq (Atezolizumab) PD-L1 inhibitor cell signaling |
| AEG 35156 | Afinitor (Everolimus) |
| Aprinocarsen | Alecensa (Alectinib) |
| Gataparsen (LY2181308) | Alunbrig (Brigatinib) |
| Oblimersen (G3139) | Gilotrif (Afatinib Dimaleate) |
| Alvocidib | Tagrisso (Osimertinib) |
| AN 9 (Pivanex) | Tarceva (Erlotinib Hydrochloride) |
| BI 2536 | Xalkori (Crizotinib) |
| Biricodar (VX-710) |  |
| BMS 214662 |  |
| Exisulind |  |
| Ilmofosine (CAS 83519-04-4) |  |
| Iniparib |  |
| Ispinesib |  |
| Lonafarnib |  |
| Mopidamol (RA-233) |  |
| Navitoclax |  |
| Obatoclax |  |
| PD 325901 |  |
| Perifosine |  |
| Rebimastat |  |
| Retaspimycin (IPI-504) |  |
| Tacedinaline (CI-994) |  |
| Talabostat |  |
| Talotrexin |  |
| Tozasertib |  |
| UCN 01 |  |
| Veliparib |  |
| Defactinib (VS-6063) |  |
| Tivantinib |  |
| CEP 9722 |  |
| Pelitinib |  |
| ASP8273 (Naquotinib) |  |
| ISIS 2503 |  |
| ISIS 5132 |  |
| Kanglaite |  |
| MKC 1 |  |
| Mitoguazone |  |
| Anatumomab mafenatox (Anti-TAG 72)/ABR-214936 apoptosis inducer cell signaling |  |
| Cixutumumab (IMC-A12) IGF-1R inhibitor cell signaling |  |
| CMB 401 (Calicheamicin) DNA inhibitor |  |
| Drozitumab apoptosis inducer cell signaling |  |
| Figitumumab (CP-751871) IGF-1R inhibitor cell signaling |  |
| Ganitumab (AMG 479) anti-IGF1R cell signaling |  |
| Linsitinib anti-IGF1R cell signaling |  |
| Litronesib mitosis inhibitor cell signaling |  |
| Matuzumab EGFR inhibitor cell signaling |  |
| Onartuzumab cell signaling |  |
| Oncolysin S (N901) cell signaling |  |
| Parsatuzumab (Anti-EGFL7) EGFR inhibitor cell signaling |  |
| Sibrotuzumab FAP inhibitor, cell signaling |  |
| Teprotumumab (R1507) anti-IGF1R cell signaling |  |
| Zalutumumab (mAb 2F8) EGFR inhibitor cell signaling |  |
| Tarextumab (OMP-59R5) cell signaling |  |
| Binetrakin IL inhibitor cell signaling |  |
| Interferon-alpha-n3 (Alferon N) cell signaling |  |
| Interleukin-2 gene therapy - Valentis cell signaling |  |
| Amuvatinib (MP-470) |  |
| Semaxanib |  |
| Vandetanib |  |
| Gefitinib |  |
| ASP8273 (Naquotinib) |  |
| **Tumor Microenvironment and VEGF agents** | |
| Failed Drugs (n=10) | Approved Drugs (n=2) |
| AE 941 | Avastin (Bevacizumab) |
| Cediranib (AZD2171) | Cyramza (Ramucirumab) |
| Ombrabulin (AVE8062) |  |
| PAN 90806 (CP-547632) |  |
| Plinabulin NPI-2358 |  |
| Pomalidomide |  |
| Tivozanib |  |
| Vadimezan (DMXAA) |  |
| Pazopanib |  |
| Volociximab antiangiogenesis |  |
| **Immunotherapy (including vaccines)** | |
| Failed Drugs (n=9) | Approved Drugs (n=0) |
| Mitumomab vaccine immunotherapy |  |
| Monoclonal antibody 2A11 (bombesin-like peptide/GRP antagonist) immunomodulator |  |
| Monoclonal antibody KS1/4-methotrexate conjugate immunomodulator |  |
| Tucotuzumab celmoleukin immunomodulator |  |
| Urelumab immunomodulator |  |
| Anti-idiotype cancer vaccine 3H1 - Titan |  |
| Cancer vaccine NY-ESO-1 - PowderMed |  |
| Cancer vaccine - Anosys |  |
| Lung cancer vaccine - GlaxoSmithKline (MAGE-A3) |  |
| **Miscellaneous** | |
| Failed Drugs (n=10) | Approved Drugs (n=0) |
| Adenosine triphosphate |  |
| Belagenpumatucel-L |  |
| INGN 225 |  |
| Marimastat |  |
| SCH 58500 |  |
| Atrasentan (ABT-627) |  |
| Contusugene ladenovec - Gendux/Introgen |  |
| DN 101 |  |
| Efaproxiral (RSR13) |  |
| Motexafin gadolinium (MGd) |  |
| Neutral metoclopramide |  |
