# Supplementary material for: A Systematic Review of the Efficacy of Preclinical Models of Lung Cancer Drugs
Source: Front Oncol. 2020 Apr 23;10:591. doi: 10.3389/fonc.2020.00591 (PMC7190806; doi:10.3389/fonc.2020.00591)
Supplement: Supplementary file 2 [file Table_2.DOCX]

**Supplemental Table 2. List of Preclinical Cell Model References.** PubMed IDs (PMID) and Digital Object Identifiers (DOI) of articles that reported preclinical in vitro cell model experiments for all approved and failed lung cancer drugs, listed by drug name.

| **Drug Name** | **PMID - Cell Model** |
| --- | --- |
| 4-Ipomeanol | 3011249 |
| Abraxane (Paclitaxel Albumin-stabilized Nanoparticle Formulation) | 26182353 |
| ABT 510 | 19139114 |
| Adenosine triphosphate | 15492806 |
| AE 941 | 12492112 |
| AEG 35156 | 16951243 |
| Alecensa (Alectinib) | 21575866 |
| Alimta (Pemetrexed Disodium) | 20548032 |
| Alunbrig (Brigatinib) | 27780853 |
| AN 9 (Pivanex) | 17267032 |
| Anatumomab mafenatox (Anti-TAG 72)/ABR-214936 | 24445502 |
| Anti-idiotype cancer vaccine 3H1 - Titan | 1698862 |
| Aprinocarsen | 15701280 |
| ASP8273 (Naquotinib) | DOI: 10.1158/1538-7445.AM2014-1728 |
| BBR 3464 (Triplatin Tetranitrate) | 10914703 |
| Belagenpumatucel-L | 11938454 |
| Biricodar (VX-710) | 9073309 |
| BMS 181174 | 7488240 |
| BMS 184476 | 15736410 |
| BMS 214662 | 11606387 |
| BMS 275183 | 11448919 |
| Cancer vaccine - Anosys | 8642258 |
| Cancer vaccine NY-ESO-1 - PowderMed | 24535937 |
| Canfosfamide (TER 286) | 29420887 |
| Cediranib (AZD2171) | 20044618 |
| CEP 9722 | 26796987 |
| Chlorosulfaquinoxaline (CQS) | 11085507 |
| Defactinib (VS-6063) | 24062525 |
| Diflomotecan (BN80915) | 11306474 |
| DMDC | 9515801 |
| DN 101 (new formulation of calcitriol, preclinical studies were done on old formulations of calcitriol) - cell lines OK, only TGI excluded | 11309356 |
| Doxorubicin Hydrochloride | 15638938 |
| Drozitumab | 27330806 |
| Efaproxiral (RSR13) | 10.1002/(SICI)1098-2299(199605)38:1<1::AID-DDR1>3.0.CO;2-O |
| Efatutazone | 18511262 |
| Epidermal growth factor fusion toxin (DAB389EGF) | 1939154 |
| Etalocib (LY293111) | 12374694 |
| Figitumumab (CP-751871) | 24107449 |
| Ganitumab (AMG 479) | 21385891 |
| Gemcitabine elaidate (CO-101 and CP-4126) | 20066470 |
| Gemzar (Gemcitabine Hydrochloride) | 16204081 |
| Gilotrif (Afatinib Dimaleate) | 26885448 |
| Glufosfamide | 27413637 |
| Ilmofosine (CAS 83519-04-4) | 15329361 |
| ILX 295501 | 14654534 |
| Imetelstat | 27192120 |
| INGN 225 | 11938454 |
| Iniparib | 22128301 |
| Interferon-alpha-n3 (Alferon N) | 15868951 |
| Interleukin-2 gene therapy - Valentis | 8795583 |
| Iressa (Gefitinib) | 11751413 |
| ISIS 2503 | 11571742 |
| ISIS 5132 | 12114419 |
| KOS 862 (Epothilone D) | 11438750 |
| Litronesib | 26304237 |
| Lometrexol | 8958184 |
| Lung cancer vaccine - GlaxoSmithKline (MAGE-A3) | 24830315 |
| Matuzumab | 18765534 |
| Mekinist (Trametinib) and tafinlar (combination) | 23438367 |
| Milataxel (MAC-321) | 14555706 |
| Mitoguazone | 9442474 |
| Monoclonal antibody 2A11 (bombesin-like peptide/GRP antagonist) | 2993906 |
| Monoclonal antibody KS1/4-methotrexate conjugate | 7834611 |
| Mopidamol (RA-233) | 8304965 |
| Motexafin gadolinium (MGd) | 15867382 |
| Mustargen (Mechlorethamine Hydrochloride) | 24274902 |
| Neutral metoclopramide | 11953831 |
| Oblimersen (G3139) | 16564596 |
| Oncolysin S (N901) | 8384975 |
| PAN 90806 (CP-547632) | 14612527 |
| Parsatuzumab (Anti-EGFL7) | 23945239 |
| Pegamotecan (Enzon) | 25987370 |
| Pelitinib (EKB-569) | 14749472 |
| Perifosine | 21496273 |
| Picoplatin | 20371711 |
| Plinabulin NPI-2358 | DOI: 10.1158/1535-7163.TARG-15-A184 |
| PR 104 | 17606726 |
| Rabusertib | 24942404 |
| Rebimastat | 20587068 |
| Rhizoxin | 3753552 |
| Rubitecan | 16205839 |
| Semaxanib | 9892193 |
| SGN 15 (BR96-Doxorubicin) | 9377565 |
| Sibrotuzumab | 26713448 |
| Tagrisso (Osimertinib) | DOI: 10.1158/1535-7163.TARG-13-A109 |
| Talotrexin | 19784838 |
| Tarceva (Erlotinib Hydrochloride) | 15166626 |
| Taxol (Paclitaxel) | 9244237 |
| Teprotumumab (R1507) monoclonal ab against IGF-1 | 24841203 |
| Tezacitabine | 8137252 |
| UCN 01 | 2656615 |
| Vosaroxin | 21252718 |
| Zalutumumab (mAb 2F8) | 15383606 |
| Zykadia (Ceritinib) | 23742252 |

**Supplemental Table 2. List of Preclinical Murine Model References.** PubMed IDs (PMID) and Digital Object Identifiers (DOI) of articles that reported preclinical murine model experiments for all approved and failed lung cancer drugs, listed by drug name.

| **Drug Name** | **PMID - Murine Model** |
| --- | --- |
| 4-Ipomeanol | 1446008 |
| Abitrexate (Methotrexate) | 27190706 |
| Abraxane (Paclitaxel Albumin-stabilized Nanoparticle Formulation) | 16489089 |
| ABT 510 | 19139114 |
| Adenosine triphosphate | 2597198 |
| AE 941 | 11964078 |
| AEG 35156 | 16951243 |
| Afinitor (Everolimus) | 17363557 |
| Alecensa (Alectinib) | 21575866 |
| Alimta (Pemetrexed Disodium) | 20548032 |
| Alunbrig (Brigatinib) | 27780853 |
| Alvocidib | 9815683 |
| AN 9 (Pivanex) | 18506586 |
| Anatumomab mafenatox (Anti-TAG 72)/ABR-214936 | 11437414 |
| Anti-idiotype cancer vaccine 3H1 - Titan |  |
| Aprinocarsen | 15701280 |
| ASP8273 (Naquotinib) | DOI: 10.1158/1538-7445.AM2014-1728 |
| Atrasentan (ABT-627) | 19717985 |
| BBR 3464 (Triplatin Tetranitrate) | 10914703 |
| Belagenpumatucel-L |  |
| BI 2536 | 17291758 |
| Binetrakin |  |
| Biricodar (VX-710) |  |
| BMS 181174 | 2380013 |
| BMS 184476 | 11567829 |
| BMS 214662 | 11606387 |
| BMS 275183 | 11448919 |
| Cancer vaccine - Anosys | 20145139 |
| Cancer vaccine NY-ESO-1 - PowderMed | 26942053 |
| Canfosfamide (TER 286) | 9635580 |
| Cediranib (AZD2171) | 26185056 |
| CEP 9722 | 17699724 |
| Chlorosulfaquinoxaline (CQS) | 8349430 |
| CMB 401 (Calicheamicin) | 8324745 |
| Defactinib (VS-6063) | 24062525 |
| Diflomotecan (BN80915) | 10383158 |
| DMDC | 2015596 |
| DN 101 (new formulation of calcitriol, preclinical studies were done on old formulations of calcitriol) - cell lines OK, only TGI excluded | 11309356 |
| Doxorubicin Hydrochloride | 15638938 |
| Drozitumab | 27330806 |
| Efaproxiral (RSR13) | 16514179 |
| Efatutazone | 18511262 |
| Enzastaurin | 16103100 |
| Epidermal growth factor fusion toxin (DAB389EGF) | 23172881 |
| Etalocib (LY293111) | 12374694 |
| Etoposide | 22131880 |
| Figitumumab (CP-751871) | 21642381 |
| Fosbretabulin (CA4P) | 17550961 |
| Ganitumab (AMG 479) | 23383308 |
| Gataparsen (LY2181308) | 21284701 |
| Gefitinib | 10815932 |
| Gemcitabine elaidate (CO-101 and CP-4126) | 20066470 |
| Gemzar (Gemcitabine Hydrochloride) | 16204081 |
| Gilotrif (Afatinib Dimaleate) | 26885448 |
| Gimeracil/oteracil/tegafur (S-1) | 22969964 |
| Glufosfamide | 17786181 |
| Ilmofosine (CAS 83519-04-4) | 1819746 |
| ILX 295501 | 9193871 |
| Imetelstat | 27192120 |
| INGN 225 | 11938454 |
| Iniparib | 22128301 |
| Interferon-alpha-n3 (Alferon N) | 10544084 |
| Interleukin-2 gene therapy - Valentis | 8795583 |
| Iressa (Gefitinib) | 10815932 |
| ISIS 2503 | 11571742 |
| ISIS 5132 | 8758918 |
| Ispinesib | 20068098 |
| Kanglaite | 25005526 |
| Keytruda (Pembrolizumab, MK-3475) | 29146734 |
| KOS 862 (Epothilone D) | 9861050 |
| Litronesib | 26304237 |
| Lometrexol | 8958184 |
| Lonafarnib | 11389071 |
| Lung cancer vaccine - GlaxoSmithKline (MAGE-A3) | 24830315 |
| Marimastat | 12405288 |
| Matuzumab | 18765534 |
| Mekinist (Trametinib) and tafinlar (combination) | 21523318 |
| Milataxel (MAC-321) | 14555706 |
| Mitoguazone | 9639872 |
| Mitumomab | 2056117 |
| Mivobulin (NSC 370147) | 2334919 |
| Monoclonal antibody 2A11 (bombesin-like peptide/GRP antagonist) | 10589749 |
| Monoclonal antibody KS1/4-methotrexate conjugate | 2784353 |
| Mopidamol (RA-233) | 8453569 |
| Motexafin gadolinium (MGd) | 11595717 |
| Navelbine (Vinorelbine Tartrate) | 10765034 |
| Navitoclax | 18519752 |
| Neutral metoclopramide | 9208905 |
| Nolatrexed | 18359701 |
| Obatoclax | 22411899 |
| Oblimersen (G3139) | 12568310 |
| Ombrabulin (AVE8062) | 17909042 |
| Oncolysin S (N901) | 9053498 |
| Opdivo (Nivolumab) | 27610613 |
| OSI 211 (liposomal lurtotecan), NX211 | 12859997 |
| PAN 90806 (CP-547632) | 14612527 |
| Parsatuzumab (Anti-EGFL7) | 23945239 |
| Pazopanib | 17164332 |
| PD 325901 | 19649202 |
| Pegamotecan (Enzon) | 9413173 |
| Pelitinib (EKB-569) | 10973323 |
| Perifosine | 21496273 |
| Picoplatin | 20371711 |
| Plinabulin NPI-2358 | 21815749 |
| Pomalidomide (CC-4047) | 16115943 |
| Portrazza (Necitumumab) IMC11 F8 | 28293124 |
| PR 104 | 17606726 |
| Rabusertib | 24114124 |
| Rebimastat | 20587068 |
| Rhizoxin | 3753552 |
| Rubitecan | 12394260 |
| SCH 58500/Contusugene ladenovec - Gendux/Introgen | 9080122 |
| Semaxanib | 9892193 |
| SGN 15 (BR96-Doxorubicin) | 10589780 |
| Sibrotuzumab | 12183436 |
| Tacedinaline (CI-994) | 9387041 |
| Tagrisso (Osimertinib) | 24893891 |
| Talabostat | 21604185 |
| Tarceva (Erlotinib Hydrochloride) | 15166626 |
| Tarextumab (OMP-59R5) | 25934888 |
| Taxol (Paclitaxel) | 26306654 |
| Taxotere (Docetaxel) | 9496387 |
| Tecentriq (Atezolizumab) | 29317734 |
| Teprotumumab (R1507) monoclonal ab against IGF-1 | 23664098 |
| Tezacitabine | 8137252 |
| Tivantinib | 20484018 |
| Tivozanib | 16982756 |
| Urelumab | 26113085 |
| Vadimezan (DMXAA) | 16944150 |
| Vandetanib ZD6474 | 12183421 |
| Veliparib | 24817481 |
| Volociximab | 18042290 |
| Vosaroxin | 22369205 |
| Xalkori (Crizotinib) | 17483355 |
| Zalutumumab (mAb 2F8) | 15383606 |
| Zykadia (Ceritinib) | 24675041 |
