# Supplementary material for: A Systematic Review of the Efficacy of Preclinical Models of Lung Cancer Drugs
Source: Front Oncol. 2020 Apr 23;10:591. doi: 10.3389/fonc.2020.00591 (PMC7190806; doi:10.3389/fonc.2020.00591)
Supplement: Supplementary file 3 [file Table_3.DOCX]

**Supplemental Figure 1. PRISMA Flow Diagram of Literature Search and Selection Process**

Other resources with data eligible for use in study analysis (n=44)

Data sources screened and assessed for eligibility (n=308)

Full-text articles with data eligible for use in study analysis (n=242)

Data sources after duplicates removed (n=308)

Data sources from other resources - drug libraries, PubChem, etc. (n=46)

Data sources from peer-reviewed articles identified through literature (n=262)

Full-text articles excluded due to reported data incompatible with our study’s variables (n=20)

Other resources excluded due to reported data incompatible with our study’s variables (n=2)
